# The Caspofungin Paradoxical Effect is a Tolerant “Eagle Effect” in the Filamentous Fungal Pathogen *Aspergillus fumigatus*

**DOI:** 10.1128/mbio.00447-22

**Published:** 2022-04-14

**Authors:** Clara Valero, Ana Cristina Colabardini, Patrícia Alves de Castro, Jorge Amich, Michael J. Bromley, Gustavo H. Goldman

**Affiliations:** a Faculdade de Ciências Farmacêuticas de Ribeirão Preto, Universidade de São Paulo, São Paulo, Brazil; b Mycology Reference Laboratory, National Centre for Microbiology, Instituto de Salud Carlos III (ISCIII), Majadahonda, Madrid, Spain; c Manchester Fungal Infection Group, Division of Evolution, Infection, and Genomics, Faculty of Biology, Medicine and Health, University of Manchester, Manchester, United Kingdom; d Antimicrobial Resistance Network, University of Manchester, Manchester, United Kingdom; Duke University Medical Center

**Keywords:** *Aspergillus fumigatus*, caspofungin, tolerance, Eagle effect, drug heterogeneity

## Abstract

Cell responses against antifungals other than resistance have rarely been studied in filamentous fungi, while terms such as tolerance and persistence are well-described for bacteria and increasingly examined in yeast-like organisms. Aspergillus fumigatus is a filamentous fungal pathogen that causes a disease named aspergillosis, for which caspofungin (CAS), a fungistatic drug, is used as a second-line therapy. Some A. fumigatus clinical isolates can survive and grow in CAS concentrations above the minimum effective concentration (MEC), a phenomenon known as “caspofungin paradoxical effect” (CPE). Here, we evaluated the CPE in 67 A. fumigatus clinical isolates by calculating recovery rate (RR) values, where isolates with an RR of ≥0.1 were considered CPE^+^ while isolates with an RR of <0.1 were classified as CPE^–^. Conidia produced by three CPE^+^ clinical isolates, CEA17 (RR = 0.42), Af293 (0.59), and CM7555 (0.38), all showed the ability to grow in high levels of CAS, while all conidia produced by the CPE^–^ isolate IFM61407 (RR = 0.00) showed no evidence of paradoxical growth. Given the importance of the calcium/calcineurin/transcription factor-CrzA pathway in CPE regulation, we also demonstrated that all Δ*crzA*^CEA17^ (CPE^+^) conidia exhibited CPE while 100% of Δ*crzA*^Af293^ (CPE^–^) did not exhibit CPE. Because all spores derived from an individual strain were phenotypically indistinct with respect to CPE, it is likely that CPE is a genetically encoded adaptive trait that should be considered an antifungal-tolerant phenotype. Because the RR parameter showed that the strength of the CPE was not uniform between strains, we propose that the mechanisms which govern this phenomenon are multifactorial.

## OBSERVATION

The “Eagle effect,” a paradoxical reduced killing of bacterial species by specific antimicrobials at concentrations above their minimum inhibitory concentration (MIC), was first described by Eagle in 1948 (1). Since then, this phenomenon has been observed in a wide range of microorganisms with different drugs. However, its underlying mechanism of action in fungi is not fully understood, and has been related to tolerance, persistence, and treatment failure (2). Drug tolerance has been extensively studied in bacterial pathogens, where it is defined as the ability of all cells of an isogenic strain to survive and even grow at low rates in the presence of drug concentrations that are greater than the MIC. The term “persistence” describes a phenomenon where only a subpopulation of cells within an isogenic strain are drug-tolerant (3).

Aspergillus fumigatus is the most important agent of fungal pulmonary infection and causes a a wide range of conditions, including chronic and allergic lung disease (chronic pulmonary and allergic bronchopulmonary aspergillosis), which affects around 8 million people worldwide, and life-threatening systemic infections (invasive aspergillosis) with more than 300,000 cases per year (4). Few antifungal agents, such as the fungicidal azoles (first-line therapy, itraconazole, posaconazole, voriconazole, and isavuconazole), amphotericin B, and the fungistatic echinocandins (caspofungin, CAS, second-line therapy) are available to treat aspergillosis while, worryingly, clinical azole resistance has been increasingly reported (5–7). While azoles inhibit the ergosterol biosynthesis pathway by directly targeting the eburicol-14-demethylase (Cyp51A/ERG11) (8), CAS acts by noncompetitively inhibiting the fungal β-1,3-glucan synthase (Fks1), which is essential for the biosynthesis of β-1,3-glucan in the fungal cell wall (9). In patients suffering from invasive aspergillosis, strains resistant to azoles are often shown to have been acquired from the environment; however, in those suffering from chronic forms of aspergillosis, resistance typically occurs during the course of infection (10). CAS resistance has been increasingly observed in *Candida* spp. and, although infrequently described, there are reports of A. fumigatus CAS resistance from patients with chronic aspergillosis (11, 12).

To date, the description of tolerance in fungi has focused almost exclusively on yeast-like fungi, where tolerance is frequently observed to occur in subpopulations within an isogenic strain, detected in some reports as a “fraction of growth” (13–15). Although there are scarce reports defining drug tolerance and persistence in filamentous fungi, one adaptive phenomenon has been reported regularly in A. fumigatus. It is known as the “caspofungin paradoxical effect” (CPE) and relies on the capacity of some clinical isolates to grow and tolerate CAS concentrations above the minimum effective concentration (MEC). Despite several existing mechanisms having already been described for A. fumigatus CPE (16, 17), there is little understanding of whether CPE occurs as a result of phenotypic heterogeneity within an isogenic population. Here, based on the characterization of CPE presence in a series of A. fumigatus clinical isolates, we demonstrate that conidia from A. fumigatus CAS-tolerant strains do not exhibit CAS heterogeneity and hence, that CPE should be considered a tolerant but not persistent phenotype.

**FIG 1:**
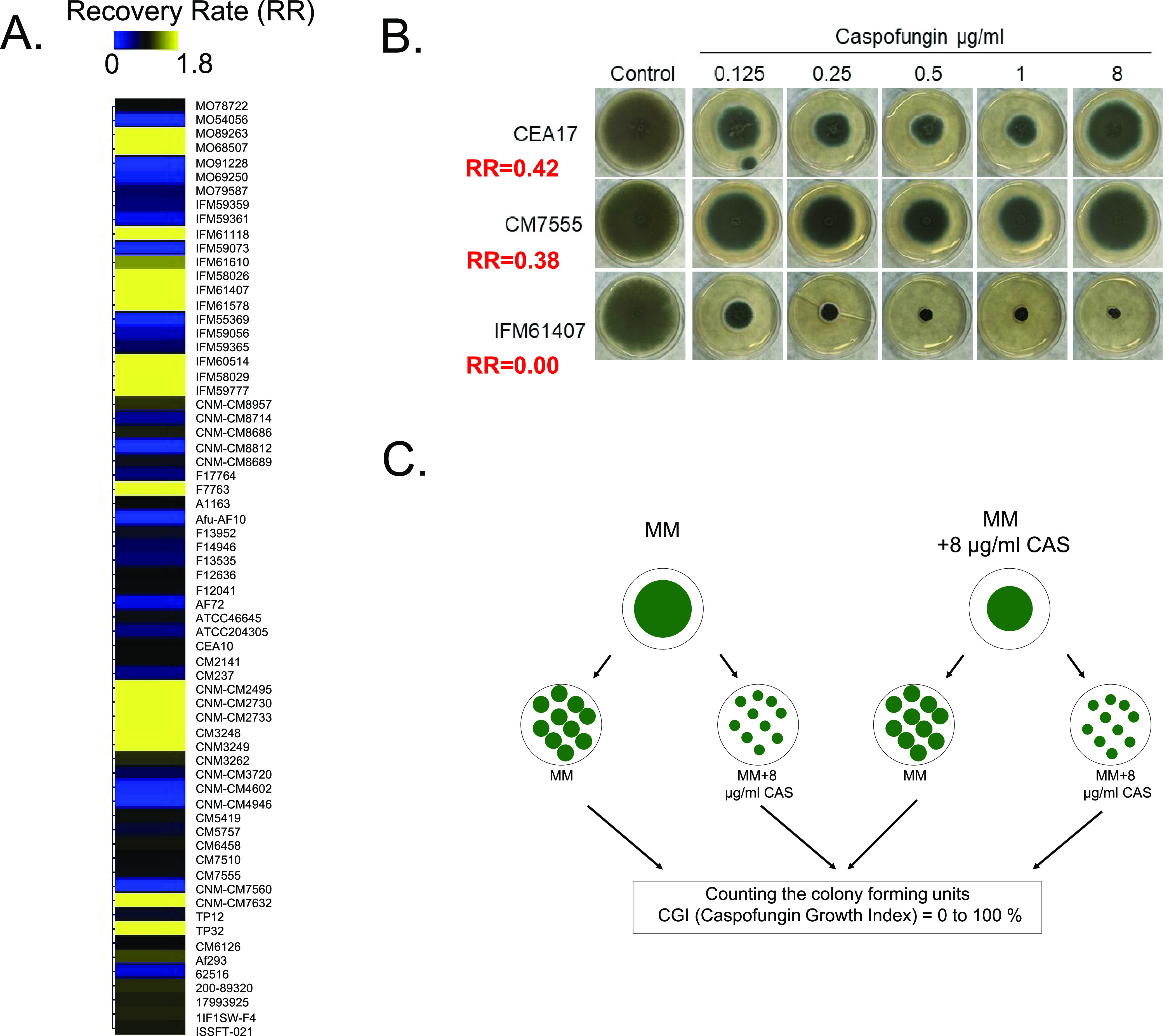
Distribution of A. fumigatus CAS tolerance in 67 clinical isolates, recovery rate values (RR) and definition of CAS growth index (CGI). (A) Heat map depicting recovery rate (RR) according to the following formula: colony diameter (8 μg/mL CAS) − minimum colony diameter/colony diameter (MM) − minimum colony diameter, where RR ≥ 0.1 isolates are CPE^+^ and RR < 0.1 isolates are CPE^–^. Heat map scale and gene identities are indicated. Hierarchical clustering was performed in MeV (http://mev.tm4.org/) using Pearson correlation with complete linkage clustering. (B) Growth of A. fumigatus CEA17, CM7555, and IFM61407 clinical isolates on MM and MM + CAS (increasing concentrations). Strains were grown for 5 days at 37°C. (C) Scheme showing how the CGI was calculated. A. fumigatus isolates were grown on MM or MM + 8 μg/mL CAS for 5 days at 37°C. Conidia were harvested in phosphate-buffered saline (PBS)-Tween 0.1%, filtered, and diluted to 10^3^ sp/mL, and 100 μL was plated in MM or MM + 8 μg/mL CAS and incubated for 2 or 3 days at 37°C. The number of colonies was counted in both treatments and CGI was determined as follows: CGI (%) = (number of colonies with radial diameter of ≥0.5 cm on MM + 8 μg/mL CAS/number of colonies radial diameter of ≥0.5 cm on MM) × 100.

We investigated CPE in 67 A. fumigatus clinical isolates (Tables S1 and S2 in the supplemental material at 10.6084/m9.figshare.19178888; S. Zhao et. al., unpublished data) by calculating the recovery rate (RR) parameter as follows: colony diameter (8 μg/mL CAS) – minimum colony diameter/colony diameter (control condition) – minimum colony diameter, where isolates with an RR of  ≥0.1 were considered CPE^+^ while isolates with an RR of <0.1 were classified as CPE^–^. Figure 1A shows a heat map representing the RR values of 67 A. fumigatus clinical isolates grown for 4 days at 37°C on minimal medium (MM)  with 0.125 to 8 μg/mL of CAS. Radial growth in the presence of CAS is exemplified for three clinical isolates: CEA17/A1163 (RR = 0.42), CM7555 (RR = 0.38), and IFM61407 (RR = 0.00) (Fig. 1B).

**FIG 2:**
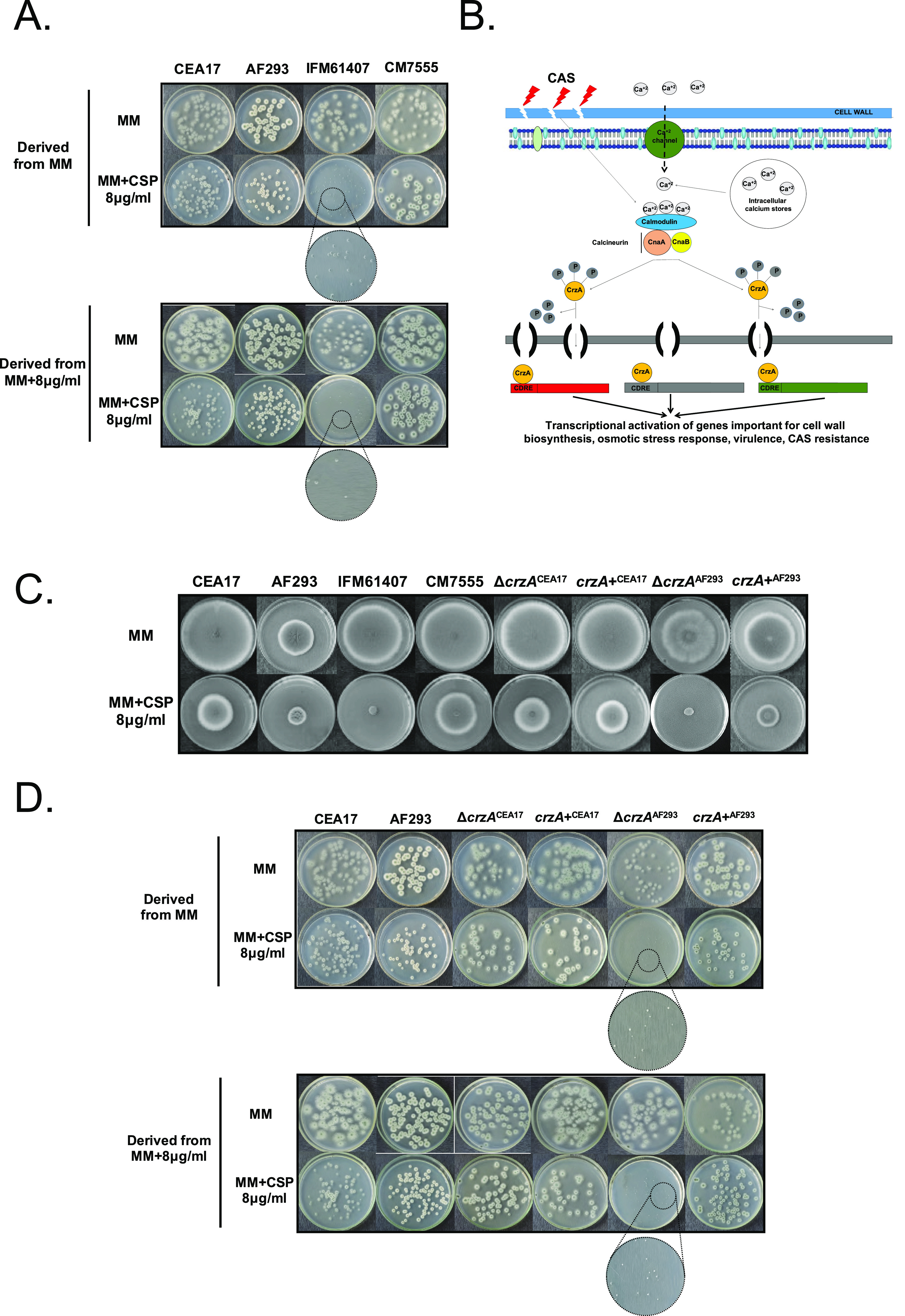
CAS growth index for A. fumigatus clinical isolates. (A) CEA17, Af293, CM7555, and IFM61407 clinical isolates were grown on MM and MM + 8 μg/mL CAS for 5 days at 37°C. Conidia were harvested in PBS-Tween 0.1%, filtered, and diluted to 10^3^ sp/mL, and 100 μL was plated in MM or MM + 8 μg/mL CAS and incubated for 2 or 3 days at 37°C. The number of colonies was counted in both treatments and the CGI was determined. (B) Scheme showing the calcium/calcineurin/CrzA pathway. Upon cell wall damage by CAS, calcium concentrations increase in the cytoplasm by calcium transport or mobilization of endogenous calcium deposits. Calcium binds to calmodulin, activating calcineurin, which directly dephosphorylates CrzA, resulting in its translocation to the nucleus. CrzA binds to calcineurin-dependent response element promoters, activating the transcriptional programs that promote stress tolerance. (C) CEA17, Af293, Δ*crzA*^CEA17^, and Δ*crzA*^Af293^ strains were grown on MM and MM + CAS for 5 days at 37°C. (D) Δ*crzA*^CEA17^ and Δ*crzA*^Af293^ strains were grown on MM and MM + CAS for 5 days at 37°C. Conidia were harvested in PBS-Tween 0.1%, filtered, and diluted to 10^3^ sp/mL, and 100 μL was plated on MM or MM + 8.0 μg/mL CAS and incubated for 2 or 3 days at 37°C. The number of colonies was counted in both treatments and the CGI was determined.

A. fumigatus sexual and asexual spores are the single developmental “cell-like” structures with a single nucleus in the fungus. Germlings and mycelia are syncytia with several nuclei present in a common cytoplasm. Is the CPE present in a “fraction” of the conidial population or in every single conidium in a single CPE^+^ clinical isolate? To address this question, we grew two A. fumigatus reference isolates, CEA17/A1163 (RR = 0.42) and Af293 (CPE = 0.59), in MM in the presence or absence of CAS 8 μg/mL for 4 days at 37°C. Then, conidia were harvested in phosphate-buffered saline (PBS)-Tween 0.1%, filtered, and diluted to 10^3^ conidia/mL, and 100 μL was plated on MM (control) or on MM + 8 μg/mL CAS (CPE concentration) (Fig. 1C). After 48 h (MM) or 72 h (MM + CAS) of growth at 37°C, the number of colonies with a radial diameter of ≥0.5 cm was counted (Fig. 1C) and the CAS growth index (CGI) was determined according to the following formula: % = (number of colonies with radial diameter of ≥0.5 cm on MM + 8.0 μg/mL CAS/number of colonies with radial diameter of ≥0.5 cm on MM) × 100 (Fig. 1C). When CEA17, Af293, and CM7555 clinical isolates were grown on either MM or MM + 8.0 μg/mL CAS, we observed a CGI of 100% (Fig. 2A, see Table S3 at 10.6084/m9.figshare.19178888). We did not observe radial diameter size heterogeneity in any of the colonies grown on MM or MM + 8 μg/mL CAS (all were > 0.5 cm radial diameter; Fig. 2A). These results indicate that every single conidium in A. fumigatus CPE^+^ strains was intrinsically able to grow at CPE CAS concentrations. We then evaluated the CGI for the clinical isolate IFM61407 (CPE^–^) (Fig. 1C). IFM61407 conidia derived from MM or MM + 8.0 μg/mL CAS both showed a CGI of 0% (Fig. 2A, Table S3 at 10.6084/m9.figshare.19178888).

Calcium homeostasis has been reported to play a central role in the CPE cellular response in A. fumigatus (18, 19) (Fig. 2B). CAS increases the intracellular calcium (Ca^2+^) concentration, activating the calcineurin-CrzA pathway (20). CrzA regulates the activation of several stress responses and cell-wall modifications (19, 21). Interestingly, *crzA* deletion in the clinical strain Af293 results in CPE loss (22) (Fig. 2C), while *crzA* deletion in the CEA17 background results in CPE maintaining (19, Fig. 2C), demonstrating intraspecies differences or CPE heterogeneity. Unlike the Δ*crzA*^CEA17^ mutant, the Δ*crzA*^Af293^ mutant cannot activate cell-wall remodeling genes upon CAS exposure, affecting its CPE (23). The CGIs for the Δ*crzA*^CEA17^ and Δ*crzA*^Af293^ mutant strains are 100 and 0%, respectively, when the strains were grown on MM + 8.0 μg/mL CAS independently if the conidia were derived from MM or MM + 8.0 μg/mL CAS (Fig. 2D, Table S3 at 10.6084/m9.figshare.19178888). Taken together, these results indicate that the transcription factor CrzA, whose deletion results in heterogeneity in the response of the CEA17 and Af293 strains to CAS, does not show CPE heterogeneity, since all the conidia from the CPE^–^ Δ*crzA*^Af293^ strain were CPE^–^, while all the conidia from the CPE^+^ Δ*crzA*^CEA17^ strain were CPE^+^ (Fig. 2D).

Our results emphasize the view that every single conidium in an A. fumigatus CPE^+^ strain is able to grow and tolerate CPE CAS concentrations. In contrast, conidia from A. fumigatus strains which lacked CPE showed no evidence of paradoxical growth, strongly suggesting that there are no A. fumigatus CAS-tolerant subpopulations. As a conclusion, A. fumigatus CPE is a homogeneous trait within an isogenic population and should be considered an antifungal-tolerant phenotype, while CPE heterogeneity exists between strains, indicating a multifactorial origin.
